# Optimising Land-Sea Management for Inshore Coral Reefs

**DOI:** 10.1371/journal.pone.0164934

**Published:** 2016-10-20

**Authors:** Ben L. Gilby, Andrew D. Olds, Rod M. Connolly, Tim Stevens, Christopher J. Henderson, Paul S. Maxwell, Ian R. Tibbetts, David S. Schoeman, David Rissik, Thomas A. Schlacher

**Affiliations:** 1 School of Science and Engineering, University of the Sunshine Coast, Maroochydore DC, 4558, Queensland, Australia; 2 Australian Rivers Institute—Coasts and Estuaries, School of Environment, Griffith University, Gold Coast, 4222, Queensland, Australia; 3 School of Chemical Engineering, University of Queensland, St Lucia, 4072, Queensland, Australia; 4 Healthy Waterways, Level 4, 200 Creek Street, Spring Hill, 4004, Queensland, Australia; 5 School of Biological Sciences, University of Queensland, St Lucia, 4003, Queensland 4072, Australia; 6 National Climate Change Adaptation Research Facility, Griffith University, Gold Coast 4222, Queensland, Australia; Universidade Federal do Rio de Janeiro, BRAZIL

## Abstract

Management authorities seldom have the capacity to comprehensively address the full suite of anthropogenic stressors, particularly in the coastal zone where numerous threats can act simultaneously to impact reefs and other ecosystems. This situation requires tools to prioritise management interventions that result in optimum ecological outcomes under a set of constraints. Here we develop one such tool, introducing a Bayesian Belief Network to model the ecological condition of inshore coral reefs in Moreton Bay (Australia) under a range of management actions. Empirical field data was used to model a suite of possible ecological responses of coral reef assemblages to five key management actions both in the sea (e.g. expansion of reserves, mangrove & seagrass restoration, fishing restrictions) and on land (e.g. lower inputs of sediment and sewage from treatment plants). Models show that expanding marine reserves (a ‘marine action’) and reducing sediment inputs from the catchments (a ‘land action’) were the most effective investments to achieve a better status of reefs in the Bay, with both having been included in >58% of scenarios with positive outcomes, and >98% of the most effective (5^th^ percentile) scenarios. Heightened fishing restrictions, restoring habitats, and reducing nutrient discharges from wastewater treatment plants have additional, albeit smaller effects. There was no evidence that combining individual management actions would consistently produce sizeable synergistic until after maximum investment on both marine reserves (i.e. increasing reserve extent from 31 to 62% of reefs) and sediments (i.e. rehabilitating 6350 km of waterways within catchments to reduce sediment loads by 50%) were implemented. The method presented here provides a useful tool to prioritize environmental actions in situations where multiple competing management interventions exist for coral reefs and in other systems subjected to multiple stressor from the land and the sea.

## Introduction

In coastal marine environments, urbanisation, habitat loss, fishing, sediments, and pollutant inputs (e.g. wastewater) degrade ecosystem condition [1, 2]. Judicious and efficient conservation strategies are, therefore, needed to maintain the condition and functioning of such systems [3], especially as funding limitations often restrict the capacity of management agencies to comprehensively address all threats. Achieving maximum benefit from management interventions requires that management actions be prioritised, preferably in a quantitative manner, with the management actions having greatest ecological benefit implemented first. Ideally, effective prioritisation of actions will result in net benefits that are greater than the sum of individual actions (i.e. synergistic effects) [4–6]. The likelihood and scale of any such synergistic outcomes are, however, rarely quantified [7].

Marine spatial planning integrates multiple forms of management interventions that are done both in the sea (e.g. fishing restrictions or habitat restoration), and on land (e.g. reducing catchment erosion and other runoff) (e.g. [8]). For example, it is well established that no-take marine reserves work best when they are implemented together with other management interventions that aim to reduce other external impacts (e.g. eutrophication, sediments). For coral reefs, assessments of the efficacy of management actions have been mostly conducted in reef systems with relatively lower impacts from terrestrial sources [9, 10]. By contrast, inshore reefs situated within coastal embayments or estuaries are subjected to several threats from the adjacent land and catchments. Consequently, these are excellent model systems for testing how different management actions on land and in the nearshore zone will benefit inshore marine systems because: 1) the response of reefs to stressors (especially sediments, nutrients and fishing) is generally well understood, 2) management actions to mitigate the effects of stressors are widely implemented (e.g. fishing restrictions, wastewater treatment), and 3) although effects might vary between ecosystems, key threats and their ecological effects are broadly comparable to those affecting other nearshore ecosystems (e.g. oyster reefs, kelp forests, seagrass beds), imparting generality within a broader environmental management context [2, 11].

In this study, we use a Bayesian belief network (BBN) incorporating empirical ecological data of ecosystem components and processes to determine which combinations of different interventions might have the greatest influence on the ecological condition of coral reefs within broader Moreton Bay, central eastern Australia (as opposed to the scale of individual reefs). BBNs are directed graphical models that illustrate causative links between nodes (i.e. variables) via arcs (i.e. arrows). BBNs are mathematically simple, and easy to interpret, thereby allowing increased easy of interpretation, greater scrutiny and simpler interpretation by practitioners [6].

Our principal objective is to develop a tool that is useful to prioritise management actions by modelling the potential ecological outcomes resulting from different combinations in the number and type of management actions. We show that synergistic benefits of multiple interventions are difficult to achieve, so management should focus on prioritising the implementation of actions that maximise combined ecological benefits, and consider these as separate, additive improvements to ecosystem condition. We suggest that similar approaches would be valuable for a range of different systems, but particularly inshore coral reef systems that are affected by a diversity of human threats.

## Methods

### Study System

**Fig 1:**
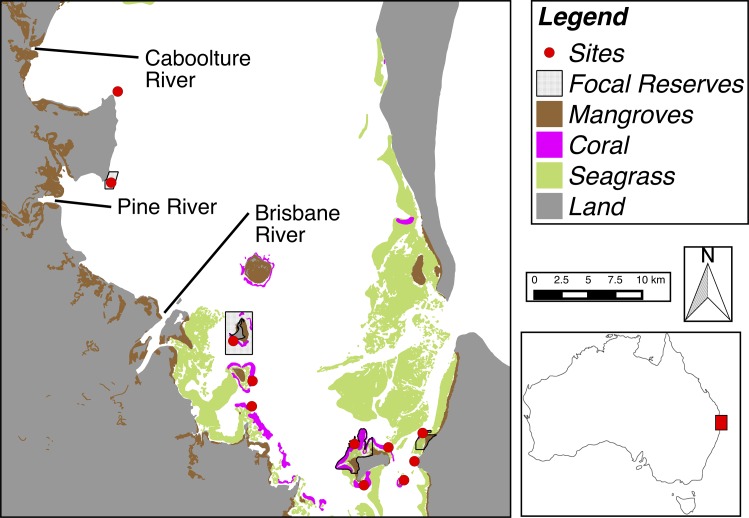
Map of study locations, ecosystem types, marine reserves, and major estuaries entering central Moreton Bay, Australia. Habitat layers courtesy Queensland Herbarium.

The inshore coral reefs of Moreton Bay in subtropical eastern Australia (27°18’S; 153°17’E, Fig 1) provide an ideal location to model the relative effectiveness of, and interaction between, multiple different approaches for ecosystem management. Moreton Bay is managed as part of a high-use marine park. It lies adjacent to the city of Brisbane (population ~2 million people increasing ~2% annually; [12]), is bounded by three barriers islands, receives input from several estuaries that drain highly-modified catchments and transport large sediment and nutrient loads to the Bay [13]. Importantly, the ecological effects of human impacts on Moreton Bay are well understood; there have been multiple, long-term (>10 years), quantitative studies that have examined how human actions alter ecological assemblages and ecosystem functions in the bay (reviewed by [14, 15, 16]).

Moreton Bay’s reefs are positioned within a shallow (<15 m), heterogeneous seascape of mangrove forests, seagrasses, and sandy and muddy seafloor [17, 18] (Fig 1). Reefs are generally dominated by massive corals, especially favids and gonioporids, and can have up to 50% cover of macroalgae [19]. Previous studies have shown that reef fish assemblages [17, 20], ecological functions [21] and MPA effectiveness [22] are influenced more by the spatial attributes of habitats within broader seascapes than by indices of water quality. Conversely, water clarity and water column nutrient concentrations regulate the cover of macroalgae on reefs and the effect of herbivory on algae is highly variable (both spatially and temporally) and low compared with tropical reefs [21, 23]. Consequently, water quality metrics (especially water column nutrient concentration and water clarity) have varied effects on reef health in Moreton Bay. These factors do not correlate with fish community structure, but correlate strongly with macroalgae, and therefore, benthic community structure.

Relatively nutrient poor and clear oceanic water enters the bay via two passages formed between barrier islands in the north and east (Fig 1). In the west, several estuaries drain a total catchment area of 22,700 km^2^ (Fig 1). Catchments contain large areas of grazing pastures (35%) and urban areas (7%), resulting in significant levels of catchment-derived sediment and nutrient entering the bay from channel erosion [13, 24]. Variable riverine runoff causes significant alterations to both benthic and pelagic community structure throughout the region over seasonal scales [20, 25].

Since the late 1990s, wastewater treatment plants have been upgraded to biological nutrient removal and this has resulted in significant reductions of effluent releases to the bay [26–28]. Treated sewage effluent does, however, still contribute 90% of point-source derived nutrients to the Bay [29].

Currently, 31% of Moreton Bay’s coral reefs are protected by no-take marine reserves, which achieves the 30% representative habitat protection targets recommended by the World Parks Congress [30]. Some studies, however, recommend up to 50% of total protection for the marine environment, particularly for systems and habitats of significance like the subtropical reefs of Moreton Bay [31, 32].

### Development of Conceptual Diagrams

**Fig 2:**
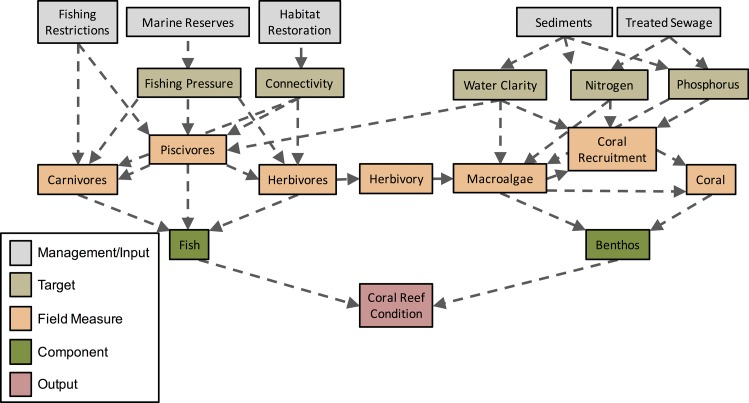
Conceptual diagram outlining the relationship between management actions and various components linked to coral reefs in Moreton Bay.

We constructed a conceptual model based on our current understanding of the *causative* relationships between management actions (i.e. a management technique that can be altered in scope or focus) and ecosystem components within Moreton Bay (Fig 2). Our conceptual framework can be divided into five levels: 1) management actions; 2) specified and measurable outcomes of management actions (target nodes); 3) quantifiable components of ecosystems, including ecological functions (field measure nodes); 4) the ecosystem components of the fish or benthic assemblages (component nodes); and 5) coral reef condition, a measure of ecosystem health, where good coral reef health is defined as a reef dominated by scleractinian corals, with low macroalgal cover (as has been the case historically in Moreton Bay [33]) and a high abundance of a diverse array of coral reef fishes (output node). S1 Table provides justifications and sources for all links between nodes.

The model comprises five levels: 1) management interventions (input/management nodes); 2) measurable outcomes of management (target nodes); 3) quantifiable components of ecosystems (field measure nodes); 4) fish or benthic assemblages (component nodes); and 5) overall coral reef condition (output node) for inshore reefs in Moreton Bay, Australia (cf. S1 Table for justifications of links between nodes for this systems).

### Construction and Testing of the Bayesian Belief Network Model

The conceptual framework was tested using a Bayesian Belief Network (BBN) in Netica v5.12 [34]. For detailed descriptions of BBN analyses and theories see [35]. Relationships between nodes for target, field measure, component and output nodes were calculated in Netica using published ecological data for 11 reefs in Moreton Bay over two seasons (22 total cases; see S2 Table), with each node containing high and low states. Given that the network was calculated using information from multiple cases across Moreton Bay, conclusions made are generalised to the scale of all reefs across the bay, rather than to specific reef sites. An added benefit of this approach is that our model incorporates all likely environmental and biological conditions present on reefs within Moreton Bay within a year, making conclusions broadly applicable at the scale of whole-of-system management.

The five management nodes that we tested were: 1) fishing restrictions in the form of bag and size limits; 2) spatial extent of no-take marine reserves; 3) restoration of habitats within the bay; 4) levels of catchment-derived sediments; and 5) discharge levels from sewage treatment plants (Table 1; for further justifications see S1 Appendix). We implement a suite of *possible* management scenarios for each management node that are calculated relative to current and historic levels of management. Consequently, we implemented four actions for each management/input node through: 1) maintaining current management levels; increasing the scale of management actions (i.e. interventions) either 2) levels that are likely to be politically and socially agreeable (henceforth ‘intervention 1’), or 3) to the full levels of management scope and intensity of management interventions that, based on current scientific evidence for the bay, are likely to have the greatest benefits (henceforth ‘intervention 2’; see Table 1); or 4) reducing management actions (henceforth ‘reductions’) (Fig 2; Table 1). Management reductions were modelled to quantify effects of possible limitations to the operating budgets of management agencies, and to assess potentially worsening conditions for certain management nodes (e.g. ongoing habitat loss or increasing effluent releases with increasing population). Full justifications and explanations of underlying data for management scenarios are available in S1 Appendix. We explicitly emphasize that the range of management interventions modelled here represent a subset of all potential management interventions that could possibly be implemented and our results should be considered in this context. Notwithstanding these constraints, the scenarios presented are reasonable and plausible combinations of management actions that may occur.

**Table 1:** Summary of levels and justifications for management node inputs. Here, Intervention 1 relates to management levels that are likely to be politically and socially agreeable, and Intervention 2 relates to the full levels of management scope and intensity of management interventions that, based on current scientific evidence, are likely to have the greatest benefits. Detailed information on the selection of these levels and the data underlying the chosen percentiles is provided S1 Appendix.

Management Node	Intervention 1		Intervention 2		Reduction		Primary Justification
	% Change	Management level	% Change	Management level	% Change	Management level	
Marine reserves	50% increase	46% of coral reefs protected	100% increase	61% of coral reefs protected	50% reduction	15.5% of coral reefs protected	Representative habitat protection levels recommended by World Parks Congress [32].
Sediments	25% reduction	3175 km of catchment waterway restoration	50% reduction	6350 km of catchment waterway restoration	25% increase	3175 km of further catchment waterway degradation	Levels calculated relative to the proposed sediment control levels recommended to maintain Moreton Bay’s ecosystem health [13].
Habitat restoration	10% increase	925 ha seagrass and 1290 ha mangrove increases	20% increase	1850 ha seagrass and 2580 ha mangrove increases	10% reduction	925 ha seagrass and 1290 ha mangrove loss	Changes relative to current data relating to habitat loss and gain in the region [36, 37].
Sewage releases	20% reduction	4mg/L total nitrogen and 2.4 mg/L total phosphorus from releases	40% reduction	3mg/L total nitrogen and 1.8 mg/L total phosphorus from releases	20% increase	6mg/L total nitrogen and 3.6 mg/L total phosphorus from releases	Current standards dictate a maximum of 5mg/L total nitrogen and 3mg/L total phosphorus entering the marine environment via outflows.
Fishing restrictions	5% increase	5% increase in the abundance of targeted fish	10% increase	10% increase in the abundance of targeted fish	10% reduction	5% reduction in the abundance of targeted fish	Calculated relative to current fishing effort within Moreton Bay [38, 39].

Management decisions are rarely made on the basis of scientific decisions alone, and always incorporate social and economic constraints. Importantly, this model was not designed to incorporate all conceivable components of ecosystem management (i.e. all ecological factors *as well as* social and economic factors), but rather to evaluate the potential ecological benefits of *likely* management interventions, thereby providing managers with a set of criteria or targets to aim for.

BBNs condition calculations on assumed response ratios, so results are necessarily contingent on the response ratios selected. Altering the response ratios of the effects of management nodes on target nodes (i.e. does an x % change in a management node’s influence result in a 1:1 change in target measurements in the bay?) would have an impact on the outcome for modelled reef condition. However, these changes are mathematically simple: a 1:2 change would result in a doubling of the effect of the management node on the target node, whilst a 2:1 change would result in a halving of that effect, and so on. These effects are trivial to calculate.

To test the model, we first conducted sensitivity analyses on the BBN to determine how sensitive findings at the coral reef condition output node were to findings at all other nodes. We then tested all possible combinations of management input nodes (n = 1024) to determine their relative influence on the likelihood of obtaining a good coral reef condition.

## Results

### Increasing Marine Reserves and Reducing Sedimentation Are Most Important for Coral Reef Health

**Fig 3:**
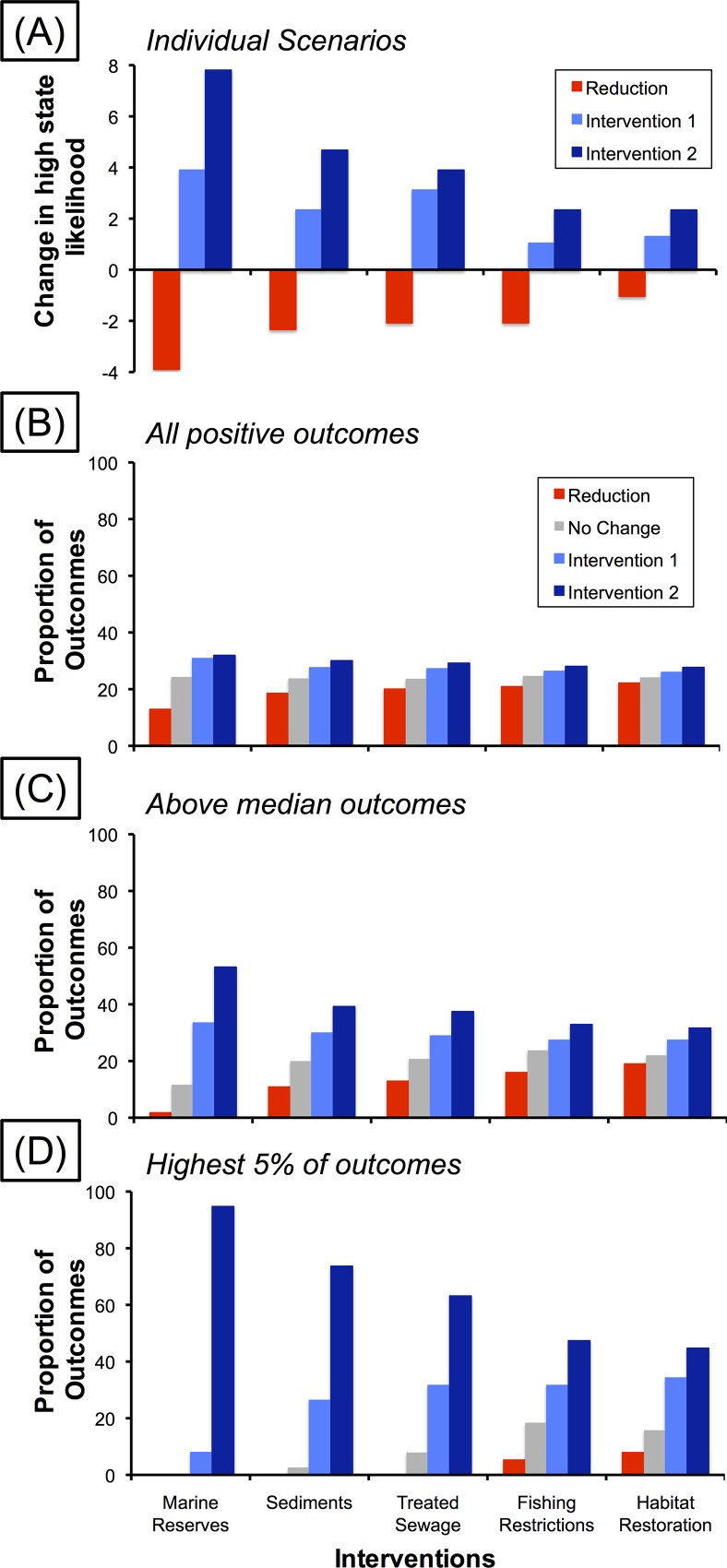
Performance of management actions: (A) likelihood of a change in state when only a single action is implemented); (B) the proportion of outcomes that resulted in positive effects on coral reef condition; (C) the proportion of outcomes that resulted in above median effects on coral reef condition; and (D) the proportion of outcomes that resulted in the highest 5% of positive effects on coral reef condition. See Table 1 for full details of each management option.

Increasing the area of marine reserves had the greatest positive effect on coral reef condition (Fig 3A). Positive effects of increasing the area of marine reserves ranked highly in most of our model outputs (Fig 3A): they were included in 63% of all outputs showing positive net benefits, in 87% of outputs where net benefits were greater than the median, and in all of the top 5% of model outputs (Fig 3B–3D). Decreasing catchment-derived sediments was the next most important management intervention for increasing the modelled condition of coral reefs (Fig 3A). The positive effects of lower sediment inputs occurred in: 58% of all scenarios with positive outcomes, 60% of above median outcomes and 98% of the highest 5% of outcomes (Fig 3B–3D). The three remaining management interventions (i.e. lower releases of treated sewage, stricter bag and length limits for recreational fishers and mangrove and seagrass restoration) were comparatively less influential compared with larger effects modelled for increasing the area of marine reserves or decreasing the volume of sediment inputs (Fig 3).

Management reductions were included in some of the combinations of management interventions that were considered to be of high ecological benefit (i.e. those in Fig 3D). These reductions, however, were only ever included for the lower ranked interventions of habitat restoration and fishing restrictions (i.e. red bars in Fig 3D). Overall, the highest-ranking suite of management interventions that contained at least one management reduction was 25% less effective than the highest modelled outcome.

**Fig 4:**
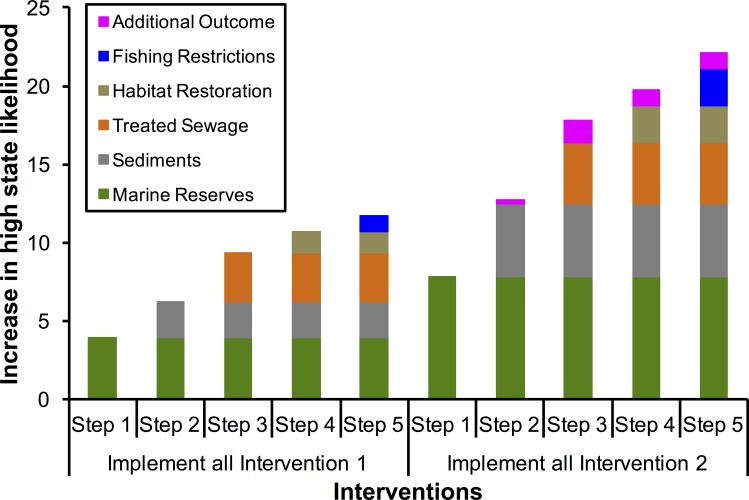
Management actions can improve the condition of inshore coral reef ecosystems in Moreton Bay, but effects are mostly additive and vary with the strategy adopted. No-take marine reserves, which prohibit fishing and increase fish biomass, are the most effective action for improving reef condition. Targeted catchment management, which reduces erosion and sediment loads, was the second-most effective action. These two actions always accounted for > 50% of improvements in reef condition, but additional gains could also be made through: improvements to wastewater treatment plants, which reduce nitrogen and phosphorus loads; restoration of other marine habitats (e.g. mangroves), which provide ecological functions that improve reef health; and focussed fishing restrictions through bag and size limits. Synergistic effects of these five management actions can result in additional improvements to reef condition, but this does not occur until the highest recommended management interventions by science have been implemented. Outcomes are displayed for two recommended management strategies (refer Table 1).

**Fig 5:**
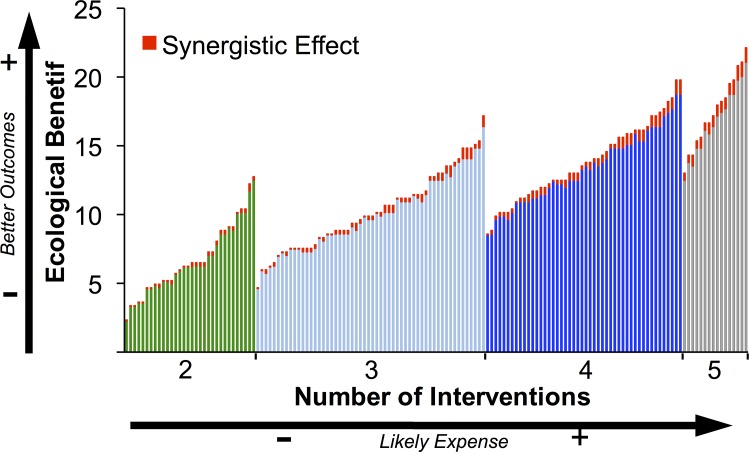
Managers often aim for synergistic effects between multiple interventions to increase 'bang for their buck' (i.e. outcome per dollar spent). Figure represents all combinations of management actions that resulted in both positive outcomes for coral reef condition, and that resulted in synergistic additions to coral reef condition (i.e. above and beyond the sum of the values of each action). Outcomes are ordered from left to right from lowest to highest modelled state of good reef condition for each number of interventions implemented. Here, the coloured ‘base' effects (green = two interventions, light blue = three interventions, dark blue = four interventions, grey = five interventions) indicates the sum of the individual interventions in isolation, and 'Synergistic Effect' indicates the additional effect of these interventions when combined.

The influence of individual management interventions on coral reef condition varied, but doubling the overall level of management interventions (i.e. implementing Intervention 2 instead of Intervention 1) always resulted in at least double the ecological benefits for coral reefs, irrespective of the particular focus of management (Fig 4; for further detail on management interventions, see Table 1). In general, when management interventions were implemented sequentially, from highest to lowest influence, their combined ecological effects on coral reef condition were mostly additive (Fig 4). Our models did not show sizeable synergistic effects on the likelihood of good coral reef condition until marine reserve extent was doubled, and sediments were halved (Fig 4). We did, however, find that synergistic effects were present in many combinations of management interventions, however, these effects were consistently below 2% (Fig 5). Models suggest that greater benefits can be potentially be achieved by implemented two management actions (reductions in sediment inputs and larger marine reserves) at greater intensity (intervention level 2) rather than implementing more management actions at a lower level of intensity (intervention 1) (Fig 4).

### Bayesian Network Less Sensitive to Lower Node Positions

In our network, modifying the values of component and field measure nodes typically had a greater influence on coral reef condition than changes to target or input/management nodes (Table 2). There were, however, several important exceptions to this pattern; varying the level of herbivory and coral recruitment (both field measure nodes) had a greater effect on benthic assemblages (a component node) and subsequent coral reef condition than did changes to fish-related component or field measure nodes. For example, the benthic component node had almost three times more influence on coral reef condition than did the fish component node, despite being located one arc higher in the network. In turn, both coral and macroalgae cover (field measure nodes) had a greater influence on coral reef condition than their counterpart fish nodes (i.e. of piscivores, herbivores and carnivores).

**Table 2:** Sensitivity analysis for output node (coral reef condition) to values at all other nodes in the Bayesian belief network. Nodes with a greater entropy reduction value have a greater influence on the outcome of the output node.

Node	Node Type	Entropy Reduction Value	Percent of max
Coral reef condition	Output	0.973	100
Benthos	Component	0.273	28
Coral	Field Measure	0.156	16
Fish	Component	0.068	6.99
Coral Recruitment	Field Measure	0.032	3.33
Macroalgae	Field Measure	0.031	3.2
Herbivores	Field Measure	0.017	1.75
Water Clarity	Target	0.017	1.72
Piscivores	Field Measure	0.016	1.66
Carnivores	Field Measure	0.01	1.03
Herbivory	Field Measure	0.009	0.943
Fishing Pressure	Target	0.007	0.759
Connectivity	Target	0.005	0.544
Nitrogen	Target	0.004	0.375
Marine Reserve extent	Input/Management	0.0009	0.092
Sediments	Input/Management	0.0003	0.032
Treated Sewage Releases	Input/Management	0.0002	0.024
Fishing Restrictions	Input/Management	0.0001	0.013
Habitat Restoration	Input/Management	0.00008	0.008
Phosphorus	Target	0.00003	0.002

## Discussion

Most ecosystems experience a range of impacts, but the capacity of managers to address all stressors is usually constrained by available resources. Consequently, it is critical that management interventions are prioritised according to their likely ecological benefits [40, 41]. Using empirical data collected from field studies of inshore coral reefs in Moreton Bay, Australia, we developed an approach for prioritising management interventions, which also identified opportunities where multiple interventions may have synergistic effects on the ecological condition of coral reefs. Our model shows that marine spatial planning will have the greatest ecological benefits for coral reefs in Moreton Bay where managers focus their investment on increasing the extent of marine reserves and simultaneously decreasing sediment loads that enter the Bay from adjacent river catchments. It is not intended to assess the efficiency, in monetary terms, of actual investment decisions. Optimising land-sea management for inshore coral reefs, therefore, requires management strategies that address impacts in both marine and terrestrial realms [41–43]. We show that synergistic benefits of multiple management interventions are unlikely to be achieved unless best recommendations from science are employed fully. The modelling approach that we developed in this study can be used to prioritize investment across the land-sea interface and ensure that impacts are addressed in the most cost effective sequence possible.

When management interventions where implemented in order of importance (i.e. from those that had the greatest to lowest influence) the ecological benefits for coral reef condition were typically additive (Fig 4). Management interventions did not have measurable synergistic effects on coral reef condition until the highest interventions recommended by science were employed for the cover of marine reserves and reductions in sediments (see Intervention 2, step 2; Fig 4). The synergistic benefits of management actions on both marine reserves and sediments were, however, consistently small (<2% improvement in modelled reef condition which is likely no greater than natural variation), compared to the significant costs required for their implementation [13, 32, 44]. In our model, synergistic benefits for coral reefs in in Moreton Bay require increasing the spatial extent of marine reserves from 31 to 62% [32], and rehabilitating 6,350 km of riparian land in adjoining catchments to reduce sediment loads (Table 1) [13]. This level of investment is probably not feasible, given other social and ecological planning considerations in the region. Nevertheless, this finding accentuates the importance of prioritising management actions to explicitly address quantitative targets and to optimise likely return on investment [7, 41]. To maximise cost effectiveness, managers might, therefore, adopt a strategy that seeks to: 1) implement interventions with the highest benefits on ecological condition up to intervention one (in this case increase marine reserves to 46% protection and reduce sediments by 25%), thereby increasing ecological outcomes for investments, 2) spend remaining moneys across the other interventions, thereby maximising the joint ecological benefits all interventions (i.e. move towards the right of Fig 5); and 3) once intervention one has been reached for all actions, seek to increase investment up to intervention two for the major actions, thereby increasing the likelihood of synergistic effects. Importantly, under such approach, it is vital for changes in management actions to be made in concert, rather than in separate pieces of legislation under different jurisdictions or organisations. This is a simple and effective approach for optimising land-sea management for coastal ecosystems, and one which might overcome much of the uncertainty that can be associated with investments in coastal conservation and ecosystem management [45–47].

Increasing the spatial extent of marine reserves and reducing sediments were the two management interventions that resulted in the greatest ecological benefits for reefs in Moreton Bay. These management interventions are important, but they can also be expensive and must be balanced against other social, economic and political considerations. It is, therefore, critical that any changes to the marine reserves or catchment rehabilitation programs are done to maximise cost-effectiveness [48–50]. Given our current understanding of factors affecting performance, it is clear that any new marine reserves should be: 1) located to maximise positive effects of connectivity (i.e. with seagrasses and mangroves) on fish assemblages and ecological functions [51]; 2) big enough to protect species with large home ranges [52]; and 3) placed at reefs that are impacted less frequently by the chronic effects of flooding, sediments and eutrophication [19, 53, 54]. Furthermore, spending money improving the condition of the catchment is important because terrestrial-based stressors can override marine-based protections (e.g. [19, 55]). Reducing catchment-born sediments is a significant challenge, both within Moreton Bay [44, 56], and more broadly [57, 58], requiring managers to focus on 1) revegetating catchment verge vegetation and rehabilitation [13, 59], 2) maintaining current levels of remnant vegetation in the catchment at the highest possible levels [60, 61], and 3) implement ideas of intelligent design of urban water runoff systems [62, 63]. Given the significant levels of catchment revegetation required to reduce loads by the scientifically recommended 50% [13], it would seem that this intervention, and associated synergistic effects between marine reserves and sediments are unlikely. Such findings surrounding the importance of controlling harvesting through marine reserves and reducing the influence of catchment-borne sediments agree with many previous articles [2, 19, 52].

Management interventions that reduced eutrophication by limiting treated sewage releases were clearly placed third to reserves and sedimentation in importance to reef health. Limiting fishing effects (through restrictions on take) and enhanced the restoration of ecologically linked habitats (e.g. mangroves and seagrass) were less important to coral reef condition than either marine reserves, sediments or sewage. However, we show that implementing management interventions which address any of these key impacts resulted in positive and quantifiable ecological benefits for coral reefs in Moreton Bay. This is crucial, as managers should not underestimate the capacity of these management actions to assist in incremental improvements in overall ecosystem condition. Further improvements will be made to sewage releases as technology advances, however, this may be offset by significant population growth within the region in future years [12], meaning that reductions in total effluent release (in terms of kg/y) are of low likelihood; a problem of global significance [64, 65]. Increasing the strictness of fishing size and bag limits is likely to be one of the more financially viable interventions discussed here, as such regulations are already policed [38]; however, such changes are likely to be politically and socially difficult to implement, and there is some uncertainty surrounding the capacity for these rules to deter fishers from reducing overall catch [66, 67] and the degree to which potential changes result in positive ecological outcomes [68, 69]. Finally, restoring up to 1850 ha and 2580 ha of seagrass and mangroves, respectively, is a significant challenge for such a small return (~2.3%), meaning that such interventions which aim only to improve coral reefs are likely financially limited. Marine habitat restoration has proven successful in many systems, but must be optimised and prioritised in much the same way that marine reserve placement is [7, 70].

Global changes (i.e. warming, sea level rise) have the potential to change and override local stressors and management [71]. Although the current study did not incorporate such effects, the long-term influences of these changes should always be considered [72]. Further, there has been extensive research conducted on the importance of microbial communities for coral reef health (e.g. [73]). Whilst microbial data is not currently available for the study system, we acknowledge that the inclusion of such data into the model might have been beneficial. In any case, the effects of such factors are likely to be lower than the impacts of our top three most includes factors.

## Conclusions

In this study, we provide model estimates of ecological benefits of management actions using empirical ecological data to show that optimising land-sea management for inshore coral reefs requires management strategies that address impacts in both marine (marine reserves) and terrestrial (sediments) realms. In our study, interventions did not have synergistic effects on coral reef condition until after maximum investment to increase the cover of marine reserves and decrease sediments; both of which are likely to be difficult to achieve both financially and politically. Synergistic benefits were also very small compared to the significant costs required for their implementation. In combination, these findings indicate that synergistic effects of multiple management actions on ecosystem condition are unlikely within our study system. Therefore, to maximise cost effectiveness in other similar systems, which have been heavily degraded by multiple human impacts, we suggest that managers should consider adopting a strategy that seeks to; 1) maximise the ecological benefits of joint ecological effects by implementing actions with the highest ecological outcomes up to intervention one first, 2) spend further moneys, up the the total managerial budget, evenly across remaining actions, and then 3) seek increasing the likelihood of synergistic effects by increasing investment up to intervention in the same order as step 1. This is a simple and effective approach for prioritizing investment across the land-sea interface for inshore coral reefs, and other coastal ecosystems that are similarly afflicted by multiple human impacts.

## Supporting Information

S1 Appendix Description and justification of modelled management interventions. (DOCX)

S1 Table Justification of all arcs between nodes in the conceptual framework for management of Moreton Bay’s reefs. (DOCX)

S2 Table Data sources for the Bayesian belief network. (DOCX)
