# Supplementary material for: Optimising Land-Sea Management for Inshore Coral Reefs
Source: PLoS One. 2016 Oct 20;11(10):e0164934. doi: 10.1371/journal.pone.0164934 (PMC5072624; doi:10.1371/journal.pone.0164934)
Supplement: S1 Appendix — (DOCX) [file pone.0164934.s001.docx]

**Appendix S1- Management scenario descriptions and justifications**

Historically, fishing restrictions in Moreton Bay have been via no-take MPAs and bag and size limits on fishers. A recent survey of recreational fishers in Queensland indicated significant recreational fishing effort within Moreton Bay and species-specific effects of recreational fishing [1]. For example, two sparid species, bream *Acanthopagrus australis* and snapper *Pagrus auratus*, dominate catches in the region. Approximately 54,000 and 27,000 individuals were harvested, for bream and snapper respectively in 2013-14 [1]. These species were abundant in our data, with bream accounting for ~12% of total counted fish and snapper accounting for ~1.5% of total counted fish. The capacity for fishing restrictions (i.e. size restrictions and bag limits) to reduce overall fishing pressure from recreational fisheries has been extensively debated, with varying conclusions [2, 3]. Therefore, we employ a conservative estimate that increased restrictions would increase piscivore and carnivore abundance by 5% and 10%, especially given the rising popularity and economic importance of recreational fisheries within Moreton Bay [4]. For the two species above, this would equate to a reduction in take of between 2,700 and 5,400 individuals for bream, and between 1,350 and 2,700 individuals for snapper within the region per year and corresponding increases in the abundance of these species at our sites [as evident from recent changes in snapper fishery restrictions within the region indicating a maximum 10% increase in fish stocks with size and bag limit restrictions; 5].

Currently, 31% of Moreton Bay’s coral reefs are protected by no-take MPAs, which achieves the 30% representative habitat protection targets recommended by the World Parks Congress (6). Some studies, however, recommend up to 50% of total protection for the marine environment, particularly for systems and habitats of significance [7, 8]. As a result, we implement 50% and 100% increases in suitably enforced MPA coverage within Moreton Bay. The 50% scenario would take MBMP part way to achieving the 50% total protection target for reefs (~47%), whilst the 100% scenario (i.e. doubling, 62%) move beyond that mark. Although changes in marine reserve coverage can be implemented fairly quickly, the effects of marine reserves on fish communities and trophic cascade recovery can take decades [9, 10], and can be site and habitat specific [11].

In our model, in-bay habitat restoration (focusing on mangroves and seagrasses) increases the level of connectivity between reefs and adjoining habitats. The current total coverage of seagrass and mangroves in Moreton Bay is approximately 18,500 ha and 25,800 ha, respectively, and there are no major restoration programs currently in place for these two habitats. Given the time and financial costs of potential restoration projects [12, 13], we implement 5 and 10% habitat restoration scenarios which equate to restoration of 925 ha and 1850 ha of seagrasses and 1290 and 2580 ha increases in mangroves, respectively. These figures of loss and gain were further supported by the relative loss of mangrove and seagrass habitats in Moreton Bay in recent decades, relative to potential habitats gain [14-16].

Studies have demonstrated that a 50% reduction in sedimentation is required to maintain the current condition of Moreton Bay [17]. Consequently, reductions in runoff quantity and velocity, and channel network revegetation are necessary for reducing overall sedimentation and associated nutrient loads. Here, a 50% reduction in sediment loads entering Moreton Bay corresponds to 36% restoration of the channel network below dams. Similarly, 50% reductions in sediment-derived nutrients require 40% an 95% catchment restoration for phosphorus and nitrogen, respectively [17]. Given these studies, we implement achievable scenarios of 25% and 50% reductions in catchment-derived sedimentation, resulting in proportionate reductions in sediment-derived nutrient concentrations. Reductions in gully erosion in the catchment, and associated reductions in sediment transport to Moreton Bay would also occur over the scale of multiple decades [17]. Indeed, fine sediments remain within inshore Moreton Bay for years following their deposition by flood events [18, 19].

Current regional standards for waste water treatment plant effluent releases result in 5mg/L total nitrogen and 3mg/L total phosphorus entering the marine environment via outflows [20]. We model scenarios where improvements in sewage processing result in a 20% and 40% reduction in TN and TP being released into the bay itself. Conversely, we model a 20% increase in sewage-derived TN and TP as a result of ongoing increases in the population of south-east Queensland [21].

Obviously, alterations to the values modelled for each scenario would result in different outcomes, however, we believe that the modelled scenarios best reflect the potential scope of management combinations for Moreton Bay. Furthermore, predicting the actual response of values at target nodes given levels of management nodes can be difficult, and potentially non-linear. For example, does an x% reduction in fishing pressure due to fishing restrictions result in an x% increase in fish communities, or does a y% reduction in nutrient outflow correspond directly to a y% reduction in sewage-borne nitrogen concentrations at our sites? The response ratios may vary in every case. Consequently, what we’re most accurately modelling here is the outcome of management interventions, rather than the actual values of the management nodes.

**References**

1. Webley J, McInnes K, Teixeira D, Lawson A, Quinn R. Statewide Recreational Fishing Survey 2013-14. Brisbane, Australia: Queensland Government, 2015.

2. van Poorten BT, Cox SP, Cooper AB. Efficacy of harvest and minimum size limit regulations for controlling short-term harvest in recreational fisheries. Fisheries Management and Ecology. 2013;20(2-3):258-67. doi: 10.1111/j.1365-2400.2012.00872.x. PubMed PMID: WOS:000316125200015.

3. Tetzlaff JC, Pine WE, III, Allen MS, Ahrens RNM. Effectiveness of size limits and bag limits for managing recreational fisheries: a case study of the Gulf of Mexico recreational gag fishery. Bulletin of Marine Science. 2013;89(2):483-502. doi: 10.5343/bms.2012.1025. PubMed PMID: WOS:000318378500005.

4. Pascoe S, Doshi A, Dell Q, Tonks M, Kenyon R. Economic value of recreational fishing in Moreton Bay and the potential impact of the marine park rezoning. Tourism Management. 2014;41:53-63. doi: 10.1016/j.tourman.2013.08.015. PubMed PMID: WOS:000328809600008.

5. Campbell AB, O'Neill MF, Sumpton W, Kirkwood J, Wesche S. Stock assessment summary of the Queensland snapper fishery (Australia) and management strategies for improving sustainability. Brisbane, Queensland: Department of Employment, Economic Development and Innovation, 2009.

6. World Parks Congress. The promise of Sydney. Sydney, Australia: 2014.

7. Dobbs K, Fernandes L, Slegers S, Jago B, Thompson L, Hall J, et al. Incorporating dugong habitats into the marine protected area design for the Great Barrier Reef Marine Park, Queensland, Australia. Ocean & Coastal Management. 2008;51(4):368-75. doi: 10.1016/j.ocecoaman.2007.08.001.

8. World Parks Congress. A strategy of innovative approaches and recommendations to reach conservation goals in the next decade. Sydney, Australia: 2014.

9. Edgar GJ, Stuart-Smith RD, Willis TJ, Kininmonth S, Baker SC, Banks S, et al. Global conservation outcomes depend on marine protected areas with five key features. Nature. 2014;506:216-20. Epub 2014/02/07. doi: 10.1038/nature13022. PubMed PMID: 24499817.

10. Claudet J, Osenberg CW, Benedetti-Cecchi L, Domenici P, García-Charton J-A, Pérez-Ruzafa Á, et al. Marine reserves: size and age do matter. Ecol Lett. 2008;11(5):481-9. doi: 10.1111/j.1461-0248.2008.01166.x.

11. Gilby BL, Stevens T. Meta-analysis indicates habitat-specific alterations to primary producer and herbivore communities in marine protected areas. Global Ecology and Conservation. 2014;2:289-99. doi: 10.1016/j.gecco.2014.10.005.

12. Spurgeon J. The socio-economic costs and benefits of coastal habitat rehabilitation and creation. Marine Pollution Bulletin. 1998;37(8-12):373-82. PubMed PMID: WOS:000082454300002.

13. Lewis RR. Ecological engineering for successful management and restoration of mangrove forests. Ecological Engineering. 2005;24(4):403-18. doi: 10.1016/j.ecoleng.2004.10.003. PubMed PMID: WOS:000230184000012.

14. Maxwell PS, Pitt KA, Olds AD, Rissik D, Connolly RM. Identifying habitats at risk: simple models can reveal complex ecosystem dynamics. Ecological Applications. 2015;25(2):573-87.

15. Manson FJ, Loneragan NR, Phinn SR. Spatial and temporal variation in distribution of mangroves in Moreton Bay, subtropical Australia: a comparison of pattern metrics and change detection analyses based on aerial photographs. Estuarine Coastal and Shelf Science. 2003;57(4):653-66. doi: 10.1016/s0272-7714(02)00405-5. PubMed PMID: WOS:000184979400011.

16. Saunders MI, Leon J, Phinn SR, Callaghan DP, O'Brien KR, Roelfsema CM, et al. Coastal retreat and improved water quality mitigate losses of seagrass from sea level rise. Global Change Biology. 2013;19(8):2569-83. doi: 10.1111/gcb.12218. PubMed PMID: WOS:000328744900023.

17. Olley J, Burton J, Hermoso V, Smolders K, McMahon J, Thomson B, et al. Remnant riparian vegetation, sediment and nutrient loads, and river rehabilitation in subtropical Australia. Hydrological Processes. 2015;29(10):2290-300. doi: 10.1002/hyp.10369. PubMed PMID: WOS:000353296900002.

18. O'Brien K, Tuazon D, Grinham A, Callaghan D. Impact of mud deposited by 2011 flood on marine and estuarine habitats in Moreton Bay. Brisbane, Australia: Griffith University, 2012.

19. Eyre B, Hossain S, McKee L. A suspended sediment budget for the modified subtropical Brisbane River estuary, Australia. Estuarine Coastal and Shelf Science. 1998;47(4):513-22. doi: 10.1006/ecss.1998.0371. PubMed PMID: WOS:000076708300011.

20. Solley D, Armstrong M. Phased upgrading for nitrogen removal - a low cost approach. Water Science and Technology. 2003;47(11):157-63.

21. Australian Bureau of Statistics, Canberra. Queensland Population Growth Canberra, Australia2012 [cited 2012 13-2-2012].
