# Supplementary material for: Optimising Land-Sea Management for Inshore Coral Reefs
Source: PLoS One. 2016 Oct 20;11(10):e0164934. doi: 10.1371/journal.pone.0164934 (PMC5072624; doi:10.1371/journal.pone.0164934)
Supplement: S1 Table — (DOCX) [file pone.0164934.s002.docx]

Table S1- Justification of all arcs between nodes in the conceptual framework for management of Moreton Bay’s reefs. ‘Rating’ refers to the certainty of information used, with 1) many recent studies highly relevant to or based in Moreton Bay, 2) studies undertaken outside of Moreton Bay, but in similar conditions, and 3) Studies in different conditions, not recent.

| **Child Node** | **Parent node** | **Functional link** | **References** | **Rating** |
| --- | --- | --- | --- | --- |
| Coral reef condition | Benthos | The benthic community contributes equally to the condition of the ecosystem, whereby a more healthy benthic community (i.e. high coral cover, low macroalgal cover) equates to a higher coral reef condition. | [1] | 2 |
|  | Fish | Fish communities contribute to coral reef condition, whereby higher abundance of a diverse array of fish equates to a higher coral reef condition, including through the provision of ecosystem services (such as nutrient cycling and herbivory) | [2, 3] | 2 |
| Benthos | Coral | Corals are the key component of benthic ecosystems on coral reefs, and compete with macroalgae for space and light, meaning that higher coral coverage equates to higher benthic health | [2, 4] | 1 |
|  | Macroalgae | Macroalgae competes directly with coral for space and light, therefore higher macroalgal cover can reduce macroalgal cover. On high macroalgal cover, inshore and back reefs, macroalgae can contribute equally to structural complexity and coral cover on reefs. | [4-6] | 1 |
| Coral | Coral Recruitment | Coral reefs rely on the successful recruitment of coral spats to continue vertical and horizontal growth | [7] | 2 |
|  | Macroalgae | Macroalgae compete directly with coral for space and light | [6] | 2 |
|  | Water Clarity | Coral growth and community structure is significantly influenced by low water clarity and sediment films | [8, 9] | 1 |
| Macroalgae | Herbivory | Herbivory controls macroalgal by top down mechanisms. In Moreton Bay, herbivores are mostly roving piscine browsers and invertebrate mesograzers | [10-12] | 1 |
|  | Nitrogen | Elevated nitrogen concentrations cause increased macroalgal coverage and biomass | [4, 13] | 1 |
|  | Phosphorus | Elevated phosphorus concentrations cause increased macroalgal coverage and biomass | [4, 13] | 1 |
|  | Water Clarity | Higher water clarity (i.e. reduced turbidity and higher secchi disc depths) increase macroalgal coverage by allowing increased photosynthetic rates | [4, 13] | 1 |
| Coral Recruitment | Macroalgae | Macroalgae impedes successful coral recruitment by preventing initial spat settlement and by shading (and killing) new recruits | [14, 15] | 3 |
|  | Nitrogen | Successful coral recruitment is limited by elevated phosphorus concentrations, likely due to increased turf algal coverage or direct chronic effects | [16] | 3 |
|  | Phosphorus | Successful coral recruitment is limited by elevated nitrogen concentrations, likely due to increased turf algal coverage or direct chronic effects | [16] | 3 |
|  | Water Clarity | Successful coral recruitment is limited by low water clarity and sediment films | [17, 18] | 2 |
| Nitrogen | Sedimentation | Nutrient input via sedimentation and associated particulate nutrients can dominate coastal ecosystems. A 50% reduction in nitrogen requires 95% restoration of remnant catchment vegetation. | [19, 20] | 1 |
|  | Treated Sewage Releases | Waste water treatment plants directly release nitrogenous waste into waterways | [21] | 1 |
| Phosphorus | Sedimentation | Nutrient input via sedimentation and associated particulate nutrients can dominate coastal ecosystems. A 50% reduction in phosphorus requires 40% restoration of remnant catchment vegetation. | [19, 20] | 1 |
|  | Treated Sewage Releases | Waste water treatment plants directly release nitrogenous waste into waterways | [21] | 1 |
| Water Clarity | Sedimentation | High sedimentation increases turbidity (c.f. nephelometric turbidity units), thereby reducing overall water clarity (cf. secchi disc measurements). A 50% reduction in sediment requires 36% restoration of remnant catchment vegetation. | [20] | 1 |
| Fish | Carnivores | The abundance of each group of fish combines to influence the overall structure of the fish community. These groups contribute equally, despite their abundances often being different, therefore, they should be applied to the fish community with equal weighting. | - | 2 |
|  | Piscivores |  | - | 2 |
|  | Herbivores |  | - | 2 |
| Carnivores | Fishing Pressure | Fishing directly reduces the abundance of piscivorous fish, especially via a moderately sized tunnel net fishery and recreational fisheries within Moreton Bay | [22, 23] | 1 |
|  | Connectivity | Connectivity (in terms of fish movement) between reefs and adjacent seagrass beds and mangrove forests increases carnivorous fish abundance on reefs in Moreton Bay | [24] | 1 |
|  | Piscivores | Piscivores prey directly on carnivores, and exert top-down control on the whole fish community | [9] | 1 |
|  | Fishing Restrictions | Fishing restrictions (i.e. maximum removal size and bag limits) reduce fishing pressure by restricting total catch of recreational fisheries, especially over short temporal scales (<years) | [25, 26] | 3 |
| Piscivores | Fishing Pressure | Fishing directly reduces the abundance of piscivorous fish, especially via a moderately sized tunnel net fishery and recreational fisheries within Moreton Bay | [22, 23] | 1 |
|  | Connectivity | Connectivity (in terms of fish movement) between reefs and adjacent seagrass beds and mangrove forests increases piscivorous fish abundance on reefs in Moreton Bay | [24, 27] | 1 |
|  | Water Clarity | Piscivores (especially visual predators) are in lower abundance in areas with higher turbidity, due to lower visual acuity | [27] | 1 |
|  | Fishing Restrictions | Fishing restrictions (i.e. maximum removal size and bag limits) reduce fishing pressure by restricting total catch of recreational fisheries, especially over short temporal scales (<years) | [25, 26] | 1 |
| Herbivores | Fishing Pressure | Fishing directly reduces the abundance of herbivorous fish, especially via a moderately sized tunnel net fishery within Moreton Bay | [22, 23] | 1 |
|  | Connectivity | Connectivity (in terms of fish movement) between reefs and adjacent seagrass beds and mangrove | [24] | 1 |
|  |  | forests increases herbivore abundance on reefs in Moreton Bay |  |  |
|  | Piscivores | Piscivores prey directly on carnivores, and exert top-down control on the whole fish community | [27] | 1 |
| Fishing Pressure | MPA Coverage | No-take marine protected areas reduces total fishing pressure (both commercial and recreational) by preventing fishing access to spatially distinct areas of the marine park | [28] | 1 |
| Connectivity | Habitat Restoration | Restoration or recovery of in-bay habitat (especially seagrasses and mangroves), increases important functional connectivity (especially in terms of fish movement) between these key habitats | [24] | 1 |

**References**

1. Hughes TP, Rodrigues MJ, Bellwood DR, Ceccarelli D, Hoegh-Guldberg O, McCook L, et al. Phase shifts, herbivory, and the resilience of coral reefs to climate change. Current Biology. 2007;17(4):360-5. doi: 10.1016/j.cub.2006.12.049. PubMed PMID: WOS:000244463100028.

2. Hughes TP, Graham NAJ, Jackson JBC, Mumby PJ, Steneck RS. Rising to the challenge of sustaining coral reef resilience. Trends in Ecology & Evolution. 2010;25(11):633-42. doi: 10.1016/j.tree.2010.07.011. PubMed PMID: WOS:000284435900005.

3. Jackson JBC, Kirby MX, Berger WH, Bjorndal KA, Botsford LW, Bourque BJ, et al. Historical overfishing and the recent collapse of coastal ecosystems. Science. 2001;293(5530):629-38. doi: 10.1126/science.1059199. PubMed PMID: WOS:000170204600040.

4. Gilby BL, Maxwell PS, Tibbetts IR, Stevens T. Bottom-up factors for algal productivity outweigh no-fishing marine protected area effects in a marginal coral reef system. Ecosystems. 2015;18(6):1056-69.

5. Evans RD, Wilson SK, Field SN, Moore JAY. Importance of macroalgal fields as coral reef fish nursery habitat in north-west Australia. Marine Biology. 2013;161(3):599-607. doi: 10.1007/s00227-013-2362-x.

6. McCook LJ, Jompa J, Diaz-Pulido G. Competition between corals and algae on coral reefs: a review of evidence and mechanisms. Coral Reefs. 2001;19(4):400-17. PubMed PMID: WOS:000169025400012.

7. Connell JH, Hughes TP, Wallace CC. A 30-year study of coral abundance, recruitment, and disturbance at several scales in space and time. Ecological Monographs. 1997;67(4):461-88. doi: 10.1890/0012-9615(1997)067[0461:aysoca]2.0.co;2. PubMed PMID: WOS:A1997YC93000003.

8. Fabricius KE. Effects of terrestrial runoff on the ecology of corals and coral reefs: review and synthesis. Marine Pollution Bulletin. 2005;50(2):125-46. doi: 10.1016/j.marpolbul.2004.11.028. PubMed PMID: WOS:000227762000014.

9. Gilby BL. Variability in marginal coral reef communities: implications for marine protected area management. Gold Coast, Australia: Griffith University; 2015.

10. Ebrahim A, Olds AD, Maxwell PS, Pitt KA, Burfeind DD, Connolly RM. Herbivory in a subtropical seagrass ecosystem: separating the functional role of different grazers. Marine Ecology Progress Series. 2014;511:83-91. doi: 10.3354/meps10901.

11. Olds AD, Pitt KA, Maxwell PS, Connolly RM. Synergistic effects of reserves and connectivity on ecological resilience. Journal of Applied Ecology. 2012;49(6):1195-203.

12. Gilby BL, Tibbetts IR, Stevens T. Low functional redundancy and high variability in Sargassum browsing fish populations in a subtropical reef system. Marine and Freshwater Research. 2016;In Press.

13. Renken H, Mumby PJ. Modelling the dynamics of coral reef macroalgae using a Bayesian belief network approach. Ecological Modelling. 2009;220:1302-14.

14. Box S, Mumby PJ. Effect of macroalgal competition on growth and survival of juvenile Caribbean corals. Marine Ecology Progress Series. 2007;342:139–49.

15. Foster NL, Box SJ, Mumby PJ. Competitive effects of macroalgae on the fecundity of the reef-building coral *Montastraea annularis*. Marine Ecology-Progress Series. 2008;367:143-52. doi: 10.3354/meps07594. PubMed PMID: WOS:000260017900012.

16. Wittenberg M, Hunte W. Effects of eutrophication and sedimentation on juvenile corals. 1. abundance, mortality and community structure. Marine Biology. 1992;112(1):131-8. doi: 10.1007/BF00349736.

17. Salinas-de-Leon P, Dryden C, Smith DJ, Bell JJ. Temporal and spatial variability in coral recruitment on two Indonesian coral reefs: consistently lower recruitment to a degraded reef. Marine Biology. 2013;160(1):97-105. doi: 10.1007/s00227-012-2066-7. PubMed PMID: WOS:000313047600009.

18. Hunte W, Wittenberg M. Effects of eutrophication and sedimentation on juvenile corals. 2. settlement. Marine Biology. 1992;114(4):625-31. doi: 10.1007/bf00357259. PubMed PMID: WOS:A1992KB47800011.

19. Eyre B, France L. Importance of marine inputs to the sediment and nutrient load of coastal-plain estuaries: a case study of Pumicestone Passage, south-eastern Queensland, Australia. Marine and Freshwater Research. 1997;48(4):277-86. doi: 10.1071/mf96071. PubMed PMID: WOS:A1997XW75800001.

20. Olley J, Burton J, Hermoso V, Smolders K, McMahon J, Thomson B, et al. Remnant riparian vegetation, sediment and nutrient loads, and river rehabilitation in subtropical Australia. Hydrological Processes. 2015;29(10):2290-300. doi: 10.1002/hyp.10369. PubMed PMID: WOS:000353296900002.

21. Pitt KA, Connolly RM, Maxwell P. Redistribution of sewage-nitrogen in estuarine food webs following sewage treatment upgrades. Marine Pollution Bulletin. 2009;58(4):573-80. doi: 10.1016/j.marpolbul.2008.11.016. PubMed PMID: 19138774.

22. Olds AD, Connolly RM, Pitt KA, Maxwell PS. Habitat connectivity improves reserve performance. Conservation Letters. 2012;5(1):56-63. doi: 10.1111/j.1755-263X.2011.00204.x. PubMed PMID: WOS:000299468900007.

23. Tibbetts IR, Townsend KA. The abundance, biomass and size of macrograzers on reefs in Moreton Bay, Queensland. Memoirs of the Queensland Museum. 2010;54(3):373-84.

24. Olds AD, Connolly RM, Pitt KA, Maxwell PS. Primacy of seascape connectivity effects in structuring coral reef fish assemblages. Marine Ecology Progress Series. 2012;462:191-203. doi: 10.3354/meps09849.

25. van Poorten BT, Cox SP, Cooper AB. Efficacy of harvest and minimum size limit regulations for controlling short-term harvest in recreational fisheries. Fisheries Management and Ecology. 2013;20(2-3):258-67. doi: 10.1111/j.1365-2400.2012.00872.x. PubMed PMID: WOS:000316125200015.

26. Bohnsack JA. A comparison of the short-term impacts of no-take marine reserves and minimum size limits. Bulletin of Marine Science. 2000;66(3):635-50. PubMed PMID: WOS:000088894300009.

27. Gilby BL, Tibbetts IR, Olds AD, Maxwell PS, Stevens T. Seascape context and predators override water quality effects on inshore coral reef fish communities. Coral Reefs. 2016;Accepted 17/3/016.

28. Marine Parks (Moreton Bay) Zoning Plan 2008, (2007).
