# Supplementary material for: Optimising Land-Sea Management for Inshore Coral Reefs
Source: PLoS One. 2016 Oct 20;11(10):e0164934. doi: 10.1371/journal.pone.0164934 (PMC5072624; doi:10.1371/journal.pone.0164934)
Supplement: S2 Table — (DOCX) [file pone.0164934.s003.docx]

| **Node Type** | **Node** | **Measure** | **Values for highest case in low state** |
| --- | --- | --- | --- |
| Target | Fishing Pressure | Current no-take MPA status (Queensland Government 2007b) | N/A (categorical variable) |
|  | Seascape Connectivity | PCA score of distance between site and nearest seagrass bed and mangrove forest (PCA1= 83% of variation explained) | 3.2 connectivity value=469m from mangroves and 2 km from seagrass |
|  | Phosphorus | TP at the site | 0.09 μm/L |
|  | Nitrogen | TN at the site | 0.09 μm/L |
|  | Water clarity | Secchi disc at the site | 4m |
| Measure- Fish | Piscivore Abundance | Abundance at the site, categorised according for FishBase | 90 individuals (Gilby 2015) |
|  | Carnivore Abundance | Abundance at the site, categorised according for FishBase | 350 individuals (Gilby 2015) |
|  | Herbivores Abundance | Abundance at the site, categorised according for FishBase | 80 individuals (Gilby 2015) |
| Measure- Process | Herbivory | PCA score of bites per minute on videoed *Sargassum* deployments bites and *Catenella* grazing rate=multiple measures and functional groups (PCA1=50.1% of variation explained) | 14% loss over 48 hours (Gilby *et al.* 2015) |
|  | Coral Recruitment | Low or high likelihood of coral recruitment success on PVC tiles from Olds *et al.* (2012c) | N/A (categorical variable) |
| Measure- Benthos | Macroalgae Cover | Coverage macroalgae at the site | 10% (Gilby *et al.* 2015) |
|  | Coral Cover | Coverage live coral at the site | 17% (Gilby *et al.* 2015) |
| Component- Fish | Fish | Standardise abundance of each fish group per case, then sum, thereby giving equal weighting to each group | 28 herbivores, 325 carnivores, 118 piscivores |
| Component- Benthos | Benthos | PCA score of all benthic categories (PCA1= 79% of variation explained) | 18% macroalgae, 14% coral cover |
| Output | Coral Reef Condition | PCA Score of benthos and fish (PCA1=54% of variation explained) | See benthic and fish component cut offs |

Table S2- Data source and entry details for the Bayesian Belief Network. We used first principle coordinates analysis scores (PCA) in R (R Core Team 2015) to determine single case values for nodes where multiple values were available (e.g. herbivory, fish/benthos; see table S1), which compress multiple values into a single factor value and weight each value equally.
